# Supplementary material for: Coordination of m6A mRNA Methylation and Gene Transcriptome in Sugarcane Response to Drought Stress
Source: Plants (Basel). 2023 Oct 24;12(21):3668. doi: 10.3390/plants12213668 (PMC10650135; doi:10.3390/plants12213668)
Supplement: Supplementary file 1 [file plants-12-03668-s001.zip › Table S1.pdf]

Table S1. The primers used for qRT-PCR.

	gene		5'--3'	Tm（℃）	Product（bp）
OAT	Sspon.01G0026110-2B	F	GAACCAGAAACTGTCCGCC	57.5	88
		R	CACTAAGCGCCTTGGATGC	59.1	
HSP70	Sspon.01G0025470-3C	F	AGGACGAGGTGGAGAGGATG	58.6	101
		R	GACTGACTCGGCTTGGTTCT	56.5	
DREB1A	Sspon.02G0009810-2B	F	GACCTCGTCGGAGCATCAC	58.1	453
		R	GCAGCGTCGTCCTCGTT	57.1	
TPI	Sspon.03G0020430-3C	F	AGGTTCCCCCTTCAGATGTTG	60.4	129
		R	CACCTCCCTTCTTCACCCA	57.7	
FBA1	Sspon.03G0000790-3D	F	TCCCTGTCCTTCTCCTTCGG	61.3	157
		R	CGGCAGCATCACCCTTGTAG	61.4	
AVP1	Sspon.04G0016680-1A	F	TGGGCTTGCTTTGGGTTACA	61.4	221
		R	CACGAATCCTGTGGCTCATC	58.4	
FKBP70	Sspon.05G0026700-1B	F	AGGGCATTACACTGGCACTC	57.4	321
		R	TCCCACTTCTCGCCTTCAAC	59.9	
GLYI-11	Sspon.06G0009730-1A	F	GGACCAGAGGACACCAACT	53.7	219
		R	TGCGAAGGCAATAACAGTGGA	61.6	
ALDH7B4	MSTRG.19319	F	TGGTGCTGAAATCGGTGGAG	61.5	283
		R	ACAACTGACGCCCATAAACGA	60.5	
GAPDH	Sspon.08G0001560-1A	F	AACGACCCCTTCATCACCAC	59.2	189
		R	ATAGTCAGCACCAGCCTCAC	54.7	
